# Editorial: Multi-omics to shed light on the pathogenesis of multifactorial diseases

**DOI:** 10.3389/fmolb.2026.1834409

**Published:** 2026-04-07

**Authors:** Giuseppina Fanelli, Anastasia Ricci, Michele Menotta, Sara Rinalducci

**Affiliations:** 1 Department of Ecological and Biological Sciences, University of Tuscia, Viterbo, Italy; 2 Department of Biomolecular Sciences, University of Urbino Carlo Bo, Urbino, Italy

**Keywords:** genes, metabolites, multifactorial diseases, multi-omics, proteins

Multifactorial diseases arise from the dynamic interplay among genetic predisposition, environmental exposures, metabolic states, immune responses, and tissue-specific regulatory networks. Within this framework, multi-omics technologies provide an integrated strategy to dissect the molecular complexity underlying disease. By combining genomics, transcriptomics, proteomics, metabolomics, and computational modeling, multi-omics approaches enable reconstruction of disease-driving molecular networks rather than isolated analysis of single factors. The five contributions collected in this Research Topic exemplify how such integrative strategies are reshaping our understanding of oncologic, inflammatory, cardiovascular, renal, and pulmonary disorders.

A first thematic axis as described by Yu et al. concerns the interaction between environmental exposure and molecular susceptibility in cancer. The study investigating endocrine disruptors (EDs) in prostate cancer integrates ADME (Absorption, Distribution, Metabolism, and Excretion) and toxicological predictions with protein–protein interaction network analysis, functional enrichment, and large-scale machine learning modeling to identify critical molecular hubs. Among them, PLK1 emerges as a central mediator through which EDs, including benzo[a]pyrene, may promote tumor progression. Molecular docking analyses (a pivotal computational technique in drug discovery that analyzes the structural and energetic interactions between ligands and target proteins) demonstrate stable binding between EDs and PLK1, while pan-cancer investigation (designed to examine similarities and differences in genomic and cellular alterations across diverse tumor types) confirms its broader oncogenic relevance. *In vitro* validation further shows that ED exposure upregulates PLK1 expression, enhancing proliferation, migration, and invasion of prostate cancer cells. Importantly, natural bioactive compounds such as cryptotanshinone effectively counteract ED-induced PLK1 activation and reverse pro-carcinogenic phenotypes. By integrating network toxicology, multi-omics profiling, computational modeling, and experimental validation, this work establishes the ED–PLK1 axis as a mechanistic bridge between environmental carcinogens and tumor progression, highlighting its potential as a chemopreventive and therapeutic target.

This Research Topic also includes a study investigating Tissue Inhibitor of Metalloproteinase 1 (TIMP1), a regulator of extracellular matrix remodeling and immune signaling, as a molecular link between oncology and cardiovascular disease (Xie et al.). Starting from transcriptomic profiling and network-based prioritization, TIMP1 is identified as a central hub gene. Subsequent pan-cancer analyses integrate expression patterns, genomic and epigenetic alterations, immune infiltration, and clinical associations across tumor types. Single-cell RNA sequencing refines its cell type–specific distribution within the tumor microenvironment. Bioinformatic findings are complemented by experimental validation in colorectal and gastric cancer models, as well as analyses in atherosclerosis and heart failure cohorts to explore cardiovascular relevance. Collectively, the study positions TIMP1 as a context-dependent regulator whose consistent overexpression in digestive cancers contrasts with distinct patterns in cardiovascular disease. This work exemplifies how multi-omics frameworks can uncover shared yet disease-specific molecular mediators at the interface of cancer and cardio-metabolic pathology.

As highlighted by Zhang et al. host–microbiota–diet interactions represent another crucial layer in chronic inflammatory disorders. The development of metagenome-informed metaproteomics (MIM) provides a methodological breakthrough in inflammatory bowel disease (IBD). By enabling simultaneous quantification of host proteins, microbial functional proteins, and dietary residues, MIM overcomes limitations of purely metagenomic approaches. The distinction between compositional dysbiosis and functional dysbiosis—detectable only at the proteomic level—demonstrates how microbial activity may diverge from genomic abundance. Moreover, combined host–bacterial protein panels show superior diagnostic performance compared to conventional biomarkers, underscoring the translational relevance of integrative proteomics. In this context, multi-omics not only clarifies pathogenic mechanisms, but also redefines diagnostic paradigms and supports precision medicine in IBD.

The importance of protein interaction networks in organ-specific disease is highlighted by the proximity-based proteomics (BioID) study mapping the Rho GTPase interactome in kidney podocytes (Ibrahim et al.). BioID enables detection of transient protein–protein interactions within intact cells, offering a near-physiological view of signaling networks. Through systematic characterization of RhoA, Rac1, and Cdc42 interactomes and integration with single-cell RNA-seq data, the study identifies novel podocyte-enriched regulators, including KIAA1522 and ARHGEF12. RhoA emerges as a dominant GTPase in podocytes, tightly controlling cytoskeletal organization and cellular integrity. ARHGEF12 is identified as a key upstream regulator of RhoA activity, essential for maintaining podocyte motility and glomerular filtration barrier function, findings validated in zebrafish models. This work illustrates how perturbations in highly specific molecular circuits translate into structural damage and clinically relevant proteinuric kidney disease.

Metabolic reprogramming constitutes another unifying theme, particularly in pulmonary fibrosis (PF). The review by Dai et al. focuses on alveolar epithelial type II (AEC2) cells, integrating transcriptomic, proteomic, and metabolomic evidence to position metabolic dysregulation as a primary driver of epithelial dysfunction and fibrotic progression. Three major metabolic axes are highlighted. Lipid metabolic alterations, together with mitochondrial and endoplasmic reticulum dysfunction, impair AEC2 homeostasis and regenerative capacity. Dysregulated carbohydrate metabolism, characterized by enhanced glycolysis, lactate accumulation, and mitochondrial dysfunction, amplifies profibrotic signaling pathways such as TGF-β. Amino acid metabolic disturbances, including glutamine and arginine pathways, further contribute to epithelial stress responses and fibrosis. Importantly, these metabolic abnormalities converge on oxidative stress, ER stress, and cellular senescence. By situating these alterations within broader regulatory networks, multi-omics approaches identify metabolism as a therapeutically actionable domain and support the development of disease-modifying strategies targeting glycolysis, mitochondrial respiration, and lipid remodeling.

Collectively, these contributions underscore three overarching principles. First, network centrality: genes, proteins, and metabolites operate within interconnected systems whose emergent properties can only be captured through integrative analyses. Second, context dependency: molecular mediators may exert distinct effects depending on tissue type, microenvironment, and disease stage. Third, translational integration: multi-omics provides a direct bridge from mechanistic discovery to biomarker development and therapeutic targeting.

Despite remarkable progress, challenges remain. Harmonization of multi-platform datasets, standardization of bioinformatic pipelines, and integration of longitudinal clinical data are essential for reproducibility. As machine learning becomes increasingly central, interpretability and experimental validation must accompany computational predictions.

This Research Topic demonstrates that multi-omics is no longer a conceptual promise, but an operational framework capable of reshaping the study of multifactorial diseases. By illuminating shared molecular nodes and disease-specific trajectories across organ systems and environmental contexts, it advances the field toward a systemic, predictive, and precision-oriented model of medicine.

